# Identification of *Barley yellow mosaic virus* Isolates Breaking *rym3* Resistance in Japan

**DOI:** 10.3390/genes15060697

**Published:** 2024-05-27

**Authors:** Hongjing Zhu, Takeshi Okiyama, Kohei Mishina, Shinji Kikuchi, Hidenori Sassa, Takao Komatsuda, Tsuneo Kato, Youko Oono

**Affiliations:** 1 Institute of Crop Science, National Agriculture and Food Research Organization (NARO), 2-1-2 Kannondai, Tsukuba 305-8602, Japan; hongjingzhu19@outlook.com (H.Z.); mishina@kca.biglobe.ne.jp (K.M.); 2 Graduate School of Horticulture, Chiba University, 648 Matsudo, Matsudo 271-8510, Japan; skikuchi@faculty.chiba-u.jp (S.K.); sassa@faculty.chiba-u.jp (H.S.); 3 Tochigi Prefectural Agricultural Experiment Station, Kawaraya-cho 1080, Utsunomiya 320-0002, Japan; okiyamat01@pref.tochigi.lg.jp (T.O.); katout10@pref.tochigi.lg.jp (T.K.); 4 Shandong Academy of Agricultural Sciences (SAAS), Crop Research Institute, 202 Gongyebei Road, Licheng District, Jinan 250100, China; takao_komatsuda@kzc.biglobe.ne.jp

**Keywords:** *Barley yellow mosaic virus*, barley, *rym3*, virus resistance, pathotype

## Abstract

In early spring 2018, significant mosaic disease symptoms were observed for the first time on barley leaves (*Hordeum vulgare* L., cv. New Sachiho Golden) in Takanezawa, Tochigi Prefecture, Japan. This cultivar carries the resistance gene *rym3* (rym; resistance to yellow mosaic). Through RNA-seq analysis, *Barley yellow mosaic virus* (BaYMV-Takanezawa) was identified in the roots of all five plants (T01–T05) in the field. Phylogenetic analysis of RNA1, encompassing known BaYMV pathotypes I through V, revealed that it shares the same origin as isolate pathotype IV (BaYMV-Ohtawara pathotype). However, RNA2 analysis of isolates revealed the simultaneous presence of two distinct BaYMV isolates, BaYMV-Takanezawa-T01 (DRR552862, closely related to pathotype IV) and BaYMV-Takanezawa-T02 (DRR552863, closely related to pathotype III). The amino acid sequences of the BaYMV-Takanezawa isolates displayed variations, particularly in the VPg and N-terminal region of CP, containing mutations not found in other domains of the virus genome. Changes in the CI (RNA1 amino acid residue 459) and CP (RNA1 amino acid residue 2138) proteins correlated with pathogenicity. These findings underscore the importance of monitoring and understanding the genetic diversity of BaYMV for effective disease management strategies in crop breeding.

## 1. Introduction

*Barley yellow mosaic virus* (BaYMV) is transmitted through soil-borne protists of the plasmodiophorid *Polymyxa graminis* [1]. BaYMV is a major pathogen causing up to 50% yield losses in susceptible barley fields worldwide; the disease is particularly severe in East Asia and Europe [2,3,4,5,6,7]. Various strains of BaYMV have been isolated globally, indicating its widespread distribution and geographical genetic diversity. Barley breeding programs have incorporated different resistance genes to combat BaYMV infection, driven by the varying pathogenicity of virus strains and the presence of other infectious viruses [8].

BaYMV is a member of the *Bymovirus* genus within the *Potyviridae* family. It exhibits distinctive filamentous particles with a diameter of about 13 nm and modal lengths of 275 and 550 nm, observed in leaf dips and partially purified virus preparations [9,10]. Its genome consists of two single-stranded, positive-sense RNA molecules: RNA1 (~7.6 kb) and RNA2 (~3.5 kb), which encode essential viral proteins crucial for replication, transcription, and pathogenicity [11]. *Potyviridae* polyproteins show a common core led by diversified leaders that are enriched in non-core modules, which expand the proteome structural and functional heterogeneity. The layout of BaYMV is leader-less RNA1 encoding the potyvirid polyprotein core and additional RNA2 [12]. RNA1, devoid of a leader sequence, encodes eight mature proteins, including P3, 6K1, CI, 6K2, NIa-VPg, NIa-pro, NIb, and CP [13]. The potyviral cylindrical inclusion (CI) protein with helicase activity for virus replication interacts directly with plasmodesmata, capsid protein-containing ribonucleoprotein complexes [14,15,16], and potyviral 6K1 protein [17] is suggested to facilitate cell-to-cell movement. The Pipo gene embedded within the P3 gene is also predicted to be in the genus *Bymovirus* and translated as a fusion protein P3N-PIPO by +2 frameshifting, facilitating cell-to-cell movement [18,19,20,21]. Potyviral VPg enhances viral RNA translation and inhibits reporter mRNA translation in planta [22]. *Bymovirus* VPg is also known to interact with the eukaryotic translation initiation factor 4E (eIF4E) through the VPg central domain during virus infection in plants [23,24,25,26]. Potyvirus CP also plays a role in cell-to-cell movement, long-distance movement, and aphid transmission [15,16]. RNA2 encodes P1 and P2 proteins, with P1 closely related to HC-pro and P2 unique among potyvirid proteins. These intricate interactions underscore the multifaceted mechanisms of BaYMV’s pathogenesis and adaptation to host plants [27,28].

BaYMV strains in Japan have evolved into distinct pathotypes, categorized as isolates I to V based on the susceptibility of barley varieties [29,30,31]. The most commonly found isolate is BaYMV-I (pathotype I) (RNA1: AB430765, RNA2: AB430766), while isolates BaYMV-II-1 (pathotype II) (RNA1: D01091), BaYMV-III (pathotype III) (RNA1: AB430767, RNA2: AB430768), BaYMV-IV (pathotype IV) (RNA1: AB430769, RNA2: AB430770), and BaYMV-V (pathotype V) (RNA1: AB450476, RNA2: AB450477) were well classified mostly in Tochigi prefecture, Japan [29,30,32,33]. Tochigi prefecture is a prominent region for two-rowed barley production in Japan, and BaYMV causes particularly serious damage to it. The virus can remain infectious for decades within the thick-walled spores produced by *P. graminis*, making it difficult to eliminate them from infected fields [8,34,35]. Therefore, planting BaYMV-resistant varieties is the only reliable control of this disease [36]. For BaYMV-resistant barley varieties, initial efforts were focused on utilizing the varieties carrying the *rym1* and *rym5* genes from cv. Mokusekko 3; after that, many resistant varieties carrying *rym5* and/or other resistance genes were bred [37]. However, pathotype III damaged resistant barley varieties with *rym5* [31]. A new cultivar, New Sachiho Golden, is developed from the high-yield and high-quality barley cv. “Sachiho Golden”, carrying *rym3* [38,39]. Increasing evidence has demonstrated that barley yellow mosaic disease is prevalent in the field at Ohtawara, Tochigi prefecture, Japan, due to the susceptibility of pathotype IV to *rym3*, while it is absent in other regions [31]. Subsequently, barley plants showing symptoms similar to barley yellow mosaic disease were also observed in a farmer’s field in Takanezawa, Tochigi prefecture, Japan. Meanwhile, the presence of BaYMV was confirmed by Western blotting analysis of collected barley plants [31]. However, to date, the virus genome in Takanezawa and the amino acid substitutions compared with the other types have not yet been studied.

To effectively manage BaYMV disease, it is crucial to conduct research on both the virus’s pathogenicity and the host’s resistance. Current breeding efforts to develop BaYMV resistance only evaluate the plant’s resistance against each BaYMV strain. This is without taking into consideration the research on virus pathogenicity, such as how BaYMV triggers infection. This study aimed to investigate the genetic factors contributing to BaYMV isolates responsible for *rym3* resistance breaking.

## 2. Materials and Methods

### 2.1. Plant Materials and Growth Conditions

The malting barley cv. New Sachiho Golden *(Hordeum vulgare* L.), which carries *rym3* (originally inherited from cv. Haganemugi), was developed at the Tochigi Prefecture Agricultural Experiment Station [38]. The Chinese landrace barley cv. Mokusekko 3, which carries *rym1* and *rym5*, is completely resistant to all BaYMV pathotypes [37]. All plants were sown in a field infected with BaYMV in Takanezawa, Tochigi Prefecture, Japan, at the end of October 2019. In mid-February 2020, five individual plants were dug up at approximately 9:00 a.m., frozen in liquid nitrogen, and stored at −80 °C until subsequent analyses.

### 2.2. Phenotypic Observation

Photos of the field plant were taken using a Canon X7 camera (Canon, Tokyo, Japan). The BaYMV-induced mosaic symptom was observed using an Axio Zoom V16 microscope (Zeiss, Oberkochen, Germany) [40].

### 2.3. RNA Extraction and Sequencing

Total RNA was extracted from infected plant root tissues using the RNeasy Mini kit (QIAGEN, Hilden, Germany) and treated with the RNase-Free DNase Set (QIAGEN, Hilden, Germany). The concentration of RNA was determined using a NanoDrop 2000 spectrophotometer (Thermo Fisher Scientific Inc., Waltham, MA, USA). RNA integrity was assessed using an Agilent 2100 Bioanalyzer (Agilent Technologies, Santa Clara, CA, USA). Five RNA-seq libraries (T01-T05) were constructed from individual plants using the TruSeq RNA Sample Preparation Kit v2 (Illumina, San Diego, CA, USA). The libraries were sequenced on the Illumina HiSeq X sequencing platform (Illumina, San Diego, CA, USA), generating 150 bp paired-end reads [41].

### 2.4. Analysis of RNA-Seq Data

Trimmomatic was used to remove adapters and low-quality bases [42]. The clean reads were aligned to the Morex genome assembly v3 [43] using HISAT [44]. To obtain unmapped reads, the samtools view (“–F4” option) and the samtools bam2fq program were used. Unmapped reads were aligned to BaYMV reference sequences, which are BaYMV of Japanese pathotype I RNA1 (AB430765) and RNA2 (AB430766) using bowtie2 [45]. Fragments aligned to those references were counted using the Linux command pipeline command, followed by the samtools view. Variant sites were identified using BCFtools [46]. SNP sites were applied if the allele frequency was ≥0.6, and the allele frequencies between >0.4 and <0.6 were called degenerate bases. The abundance of *Barley mild mosaic virus*, *Wheat yellow mosaic virus*, and *Wheat spindle streak mosaic virus* were searched by BLAST of the assembled data in the five root samples in cvs. New Sachiho Golden and Mokusekko 3. The generated RNA-seq data were deposited in the DDBJ BioProject database (accession ID. PRJDB18040).

### 2.5. Phylogenetic Analysis of BaYMV Strains

The BaYMV-I RNA1 (AB430765) and RNA2 (AB430766) nucleotide sequences were used as queries for a BlastN search against previously documented coding sequences (RNA1: Tochigi II-1 D01091, Tochigi III AB430767, Ohtawara IV AB430769, Dazhong CN DZ MW295871, Nanyang CN NY MW295868, Yancheng AJ132268, Yamaguchi V AB450476, Aschersleben ASL1 AJ515484, Germany G X69757, RNA2: Tochigi III AB430768, Ohtawara IV AB430770, Yancheng AJ132269, Yamaguchi V AB450477, Dazhong CN DZ MW295876, Nanyang CN NY MW295873, Germany D01099, Germany HYT-38 MN107380). The BaYMV-I RNA1 (BAG70349) and RNA2 (BAG70350) amino acid sequences were used as queries for a BlastX search against previously documented coding sequences (RNA1: Tochigi II-1 BAA00875, Tochigi III BAG70351, Ohtawara IV BAG70353, Yamaguchi V BBE49537, Dazhong CN DZ UWL85807, Nanyang CN NY UWL85804, Yancheng CAA10637, Germany G CAA49412, Aschersleben ASL1 CAD56476, RNA2: Tochigi III BAG70352, Ohtawara IV BAG70354, Yamaguchi V BBE49538, Yancheng CAA10638, Dazhong CN DZ UWL85812, NanYang CN NY UWL85809, Germany BAA00884, Germany HYT-38 QHA94748). The database codes of all nucleotide sequences and all amino acid sequences used in this study were deposited in GeneBank. The e-value threshold applied was 1 × 10^−50^, and the identity threshold was 90%. Query coverage of more than 90% was utilized to remove partial hits. Nucleotide sequences were aligned using the Clustal Omega algorithm [47].

### 2.6. Correlation Analysis between BaYMV Encoded Protein Residue and the Virus Infection to rym3-Carrying Barley cv. New Sachiho Golden

To analyze the responses of *rym3*-carrying barley plants against BaYMV, they were scored “−1” for resistant and “1” for susceptible [48]. The difference in the amino acid residue was scored “−1” for identical and “1” for different from the consensus sequences of BaYMV-Takanezawa-T01 DRR552862. The coefficient of determination (R^2^) is calculated between the resistance response score and the amino acid residue score. The *p*-value threshold of 0.01 is calculated from the t-score, t = r√(n − 2)/√(1 − r2), and Excel function “tdist”. For the correlation analysis, the scores (0/1 correspondence with BaYMV-Takanezawa-T01 DRR552862) of all amino acids (inferred from the RNA sequences) of different BaYMV isolates (Ohtawara IV BAG70353, Yamaguchi V BBE49537, Tochigi II-1 BAA00875, Tochigi III BAG70351, Tochigi I BAG70349, Germany G CAA49412) were compared with their capability to cause visible symptoms on the plants of barley cv. New Sachiho Golden in the Takanezawa field trial.

### 2.7. Genome-Wide Association Analysis

The R package rrBLUP [49] is used to verify correlation analysis results. The Q (population structure) + K (relative kinship) model was used to calculate the GRM (genetic relationship matrix). Minor allele frequency is set at 0.05. The FDR threshold of 0.05 was used to determine the significant peaks.

## 3. Results

### 3.1. Identification of Barley Yellow Mosaic Virus Isolates on Breaking rym3 Varieties

**Figure 1:**
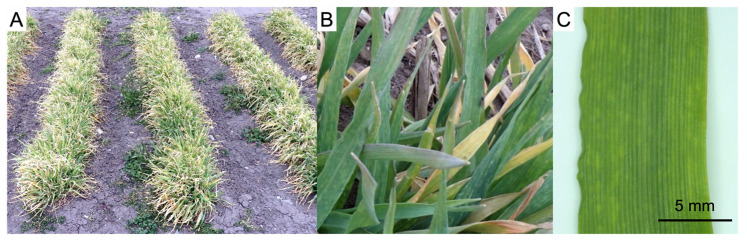
The disease symptoms of *Barley yellow mosaic virus* (BaYMV) on the cv. New Sachiho Golden in the Takanezawa field in late March 2020. (**A**) The overall appearance of the affected plants in the field. (**B**) Leaf mosaic symptoms of plants. (**C**) Observation of the second leaf from the upper new leaf.

In the autumn of 2019, cv. New Sachiho Golden was sown in the primary malting barley production fields located in Takanezawa, Tochigi prefecture, Japan (the field is hereinafter called Takanezawa). *Barley yellow mosaic virus* (BaYMV) isolates were identified on the *rym3*-carrying variety barley plant, cv. New Sachiho Golden. The leaves exhibited severe yellow mosaic symptoms at the end of February (Figure 1).

Root samples randomly selected from five individual plants grown at various parts of the same field were collected and subjected to RNA sequencing. Raw reads from 2.4 to 3.4 Gbases were obtained. From the clean reads, 81.8–87.3% were mapped to the Morex V3 genome, and the BaYMV consensus sequences were matched to Tochigi I (RNA1, AB430765; RNA2, AB430766) [50]. Quantities of BaYMV ranged from 3.8 to 6.6% in total. Subsequently, complete genome consensus sequences were obtained for RNA1 and RNA2 of the five BaYMV isolates, BaYMV-Takanezawa-T01 to BaYMV-Takanezawa-T05 (Table S1). The viral polyprotein coding sequences are consistent across all isolates and consist of the following: 7642 bp of RNA1 nucleotide sequences, which translate to 2412 aa protein; 3585 bp of RNA2 nucleotide sequences, which translate to 890 aa protein. BaYMV remained at almost zero in cv. Mokusekko 3, as a negative control. In the test of cvs. New Sachiho Golden and Mokusekko 3, several soil-borne viruses belonging to the genus *Bymovirus* (*Potyviridae*)—*Barley mild mosaic virus*, *Wheat yellow mosaic virus*, and *Wheat spindle streak mosaic virus*—were not present in these samples (Table S1).

### 3.2. The Existence of Two Distinct BaYMV Isolates in Takanezawa

The nucleotide identity among the various sequences of East Asian and European isolates ranged from 0 to 514 mismatches (93.27%) for RNA1 and up to 344 mismatches (90.40%) for RNA2 (Table S2). Amino acid identities varied from 95.69% (104 aa) to 100% for RNA1 and 96.85% (28 aa) to 100% for RNA2 (Table S3).

**Figure 2:**
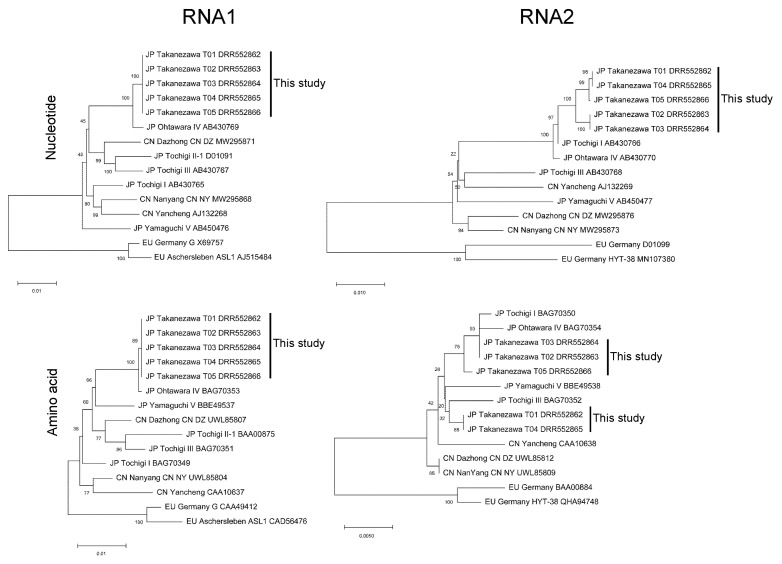
Phylogenetic tree using nucleotide and amino acid sequences of RNA1 and RNA2. A neighbor-joining phylogenetic tree was produced by the MEGA 11 program based on a ClustalW alignment of the nucleotide and amino acid sequences. The robustness of internal branches was tested using bootstrap analyses (1000 replicates).

The RNA1 of BaYMV isolates detected in Takanezawa were genetically distinct from isolates reported from China and Europe. All BaYMV-Takanezawa-T01 RNA1 to BaYMV-Takanezawa-T05 RNA1 were nearly identical. There were 40 out of 7642 sites of nucleotide variation (99.48% identity) for nucleotide sequences and 3 out of 2412 sites of variation (99.88% identity) for amino acid sequences compared with closely Japanese isolate Ohtawara IV RNA1 (AB430769) (Figure 2, Tables S2 and S3).

All five nucleotide sequences of RNA2 were highly similar to Tochigi I (BAG70350) with over 99% identity. Regarding the amino acid sequences, BaYMV-Takanezawa-T02 (DRR552863) and BaYMV-Takanezawa-T03 (DRR552864) were closely related to Ohtawara IV with 99.78% identity. In comparison, BaYMV-Takanezawa-T01 (DRR552862) and BaYMV-Takanezawa-T04 (DRR552865) were more similar to Tochigi III (BAG70352) with 99.44% identity, suggesting that two isolates of BaYMV RNA2 were identified (Figure 2, Table S3). Between BaYMV-Takanezawa-T01 (DRR552862) and BaYMV-Takanezawa-T02 (DRR552863), 22 out of 3585 sites of nucleotide variation (99.39% identity) (Table S2) and 5 out of 890 sites of amino acid variation (99.44% identity) were identified (Table S3).

### 3.3. Variable Sites of Virus Sequence in Amino Acid

**Figure 3:**
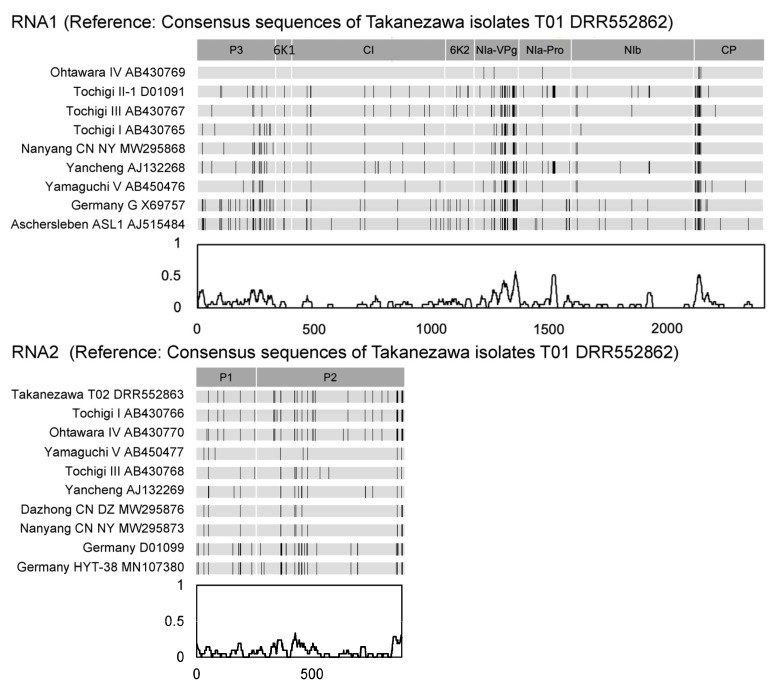
The sites of amino acid variation in the polyproteins from Barley yellow mosaic virus (BaYMV) isolates. The consensus sequences of BaYMV-Takanezawa-T01 DRR552862, RNA1, and RNA2 were used as references. Each black-colored vertical bar represents amino acid substitutions compared to the consensus sequence. Line plots at the bottom show sliding window frequency summaries in 20 aa on average.

Variable sites within the NIa-VPg, NIa-pro, and CP proteins exhibited diversification between the consensus sequence of BaYMV-Takanezawa-T01 DRR552862 and Ohtawara IV (BAG70353) (Figure 3 and Figure S1, Table S4). The most variable sites were identified in the NIa-VPg (amino acid variation of 19.79%, 37 of 187 aa) and N-terminal region of CP (amino acid variation of 7.69%, 23 of 299 aa) proteins among the East Asian and European isolates (Figure 3). These sites were related to the co-evaluation between resistance genes and the virus genome. Otherwise, permissive sites may not affect the virus’s pathogenicity.

### 3.4. Amino Acid Changes in NIa-VPg Correlated with Pathogenicity

**Figure 4:**
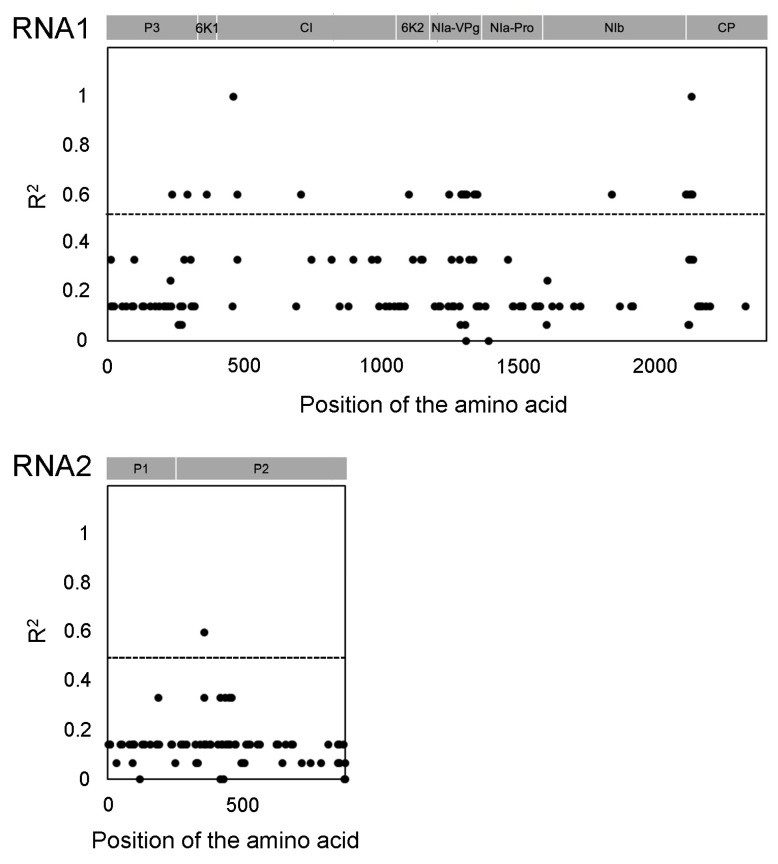
The correlation coefficient plot between the phenotypes and the sites of amino acid variations in the polyproteins. The sequences used for this analysis are summarized in Table S4. For each position of the amino acid within the protein (horizontal axis), the positions without any dots are excluded due to no correlations. The *y*-axis represents the coefficient of determination (R2) between amino acid variations and phenotypes for every site. The horizontal gray lines represent the significance thresholds for amino acid variations (*p* = 0.01 and R^2^ = 0.5).

To determine whether the amino acid changes in the newly discovered BaYMV genome are associated with breaking *rym3* resistance, we computed the correlation coefficient between the amino acid variations in RNA1 (representative BaYMV-Takanezawa-T01 DRR552862 and BaYMV-Takanezawa-T02 DRR552863) of BaYMV and the responses observed in the Takanezawa field (Figure 4 and Table S4). The VPg and the CP proteins were the most variable regions, with correlation coefficients (R^2^) greater than 0.5 and *p*-values smaller than 0.01. The changes in the CI (RNA1 amino acid residue 459) and CP (RNA1 amino acid residue 2138) proteins correlated with pathogenicity (Figure 4 and Table S5). Furthermore, one significant amino acid sequence difference was observed between polyproteins encoded by RNA2. This difference was located at amino acid residue 359, within the P2 protein. The isolates BaYMV-Takanezawa-T01 (DRR552862), Ohtawara IV (BAG70354), and Yamaguchi V (BBE49538) encoded Threonine at amino acid 359, while BaYMV-Takanezawa-T02 (DRR552863), Tochigi I (BAG70350), Tochigi III (BAG70352), and the other three Chinese (BaYMV-CN DZ UWL85812, CN NY UWL85809, BaYMV-Yancheng CAA10638) and two German (BaYMV-Germany, BAA00884, Germany HYT-38 QHA94748) isolates encoded Alanine at this position (Figure 4 and Table S4).

## 4. Discussion

### 4.1. rym3 Resistance Breaking in BaYMV-Infected Field

The resistance gene of *rym3* is commonly used in Japanese cultivars [37]. However, cultivating genotypes carrying a single resistance gene is in danger of breaking resistance under selection pressure. The *rym3* resistance has been broken in the Ohtawara and Yamaguchi fields [31]. This study provides a comprehensive understanding of barley plants’ responses to BaYMV through the RNA-Seq technique. The RNA1 sequence of the Takanezawa field is similar to that of the Ohtawara isolate. These two locations are 33 km apart but share the same RNA1 origin and are likely to be prevalent in the northern part of Tochigi prefecture. The RNA1 sequence of Yamaguchi is in a different clade than the BaYMV-Takanezawa isolates despite the two locations being 850 km apart. It is possible that the two breaking events of *rym3* resistance in Ohtawara and Yamaguchi are independent and do not share the same mutation sites in the virus genomes. It suggests that our approach was deemed useful because it was assumed that all *rym3*-breaking events corresponded to the same mutation. The *rym4* resistance-breaking strain was identified among the German isolates, and its VPg mutation was determined, but Japanese strains that break *rym4* resistance have not been identified [42]. We propose an association analysis of virus variants to identify the corresponding mutations responsible for pathogenicity changes. Our preliminary small-scale genome-wide association study analysis indicates the same site of amino acid variation in the polyprotein, suggesting that our proposal was supported (Figure 4 and Figure S2). To date, many sequences have been registered, but their pathotype response is still largely unknown. Additionally, responses of resistant genotypes in the BaYMV-infected field need further study.

### 4.2. Co-Existence Isolates of BaYMV Identified in Takanezawa

Our data revealed that at least two viruses with different genetic backgrounds of breaking resistance exist in the BaYMV-Takanezawa isolates. In virus sequence assembly, single colony isolation or RNA transcript assembly methods are typically utilized, particularly for viruses with unique sequences. We show here that, in the Takanezawa field, the analysis of RNA2 revealed the presence of a mixture of two distinct BaYMV isolates across the five biological replicates (Tables S2 and S3, and Figure S1), making it challenging to use RNA transcript assembly without producing chimeric outputs. To overcome this challenge, we use a consensus call along a reference genome. However, T05 (DRR552866) failed to reach a consensus call due to the presence of many SNPs with even allele frequencies (40–60%), resulting in degenerate bases [51]. It is worth noting that different isolates can intermix and result in the misdirection of pathotypes if treated as a single strain in the multiple pathotype field.

### 4.3. The Determinant Protein Responsible for Breaking rym3 Resistance

In this study, we examined the correlation between amino acid substitutions and disease responses. Out of 2412 amino acid residues in RNA1, 25 aa residues were candidates responsible for breaking *rym3* resistance (Table S4). Out of 890 amino acid residues in RNA2, only 1 was a candidate (Table S5). If multiple mutation events of breaking *rym3* happen, an R^2^ not equal to 1 can be assumed. Our analysis listed two amino acid residues in RNA1 with R^2^ = 1, residue 459 in CI protein, and residue 2138 in CP (Table S4). CI is involved in resistance to *Wheat yellow mosaic virus*, genus *Bymovirus*, in the family *Potyviridae* [52]. CI protein of *Soybean mosaic virus*, genus *Potyvirus*, in the family *Potyviridae*, is a pathogenic determinant provoking a lethal systemic hypersensitive response, which the function varied with its single amino acid substitution [53,54]. VPg often plays a crucial role in interacting with recessive resistance genes, while CP interacts with dominant resistance genes [55]. Previous studies have suggested that VPg is identified as a pivotal protein in overcoming *eIF4E*-mediated recessive resistance genes like *rym4/5/6* [42,56,57,58].

Our analysis listed one amino acid residue in RNA2 with an R^2^ = 0.56, residue 359 in the TMV-like coat protein (TMV-like CP) domain of the P2 protein, which is conserved in all full-length *Bymovirus* accessions except *Oat mosaic virus* [12] (Table S5). TMV-like CP sequences of bymoviruses cluster within a monophyletic clade, suggesting their common origin. The CP of family *Virgaviridae* and *Benyviridae* members shows homology with *Bymovirus* P2 [12,51]. A recent study reveals that a single amino acid substitution mutation of WYMV P2 affects the function of its viral suppressor of RNA silencing activity and its own protein stability [59]; however, P2 protein biochemical properties and function are still largely unknown. We require more sequences and *rym3* responses to perform a correlation analysis. Although many isolates are currently registered, the response to different pathotypes remains to be further studied. It would also be useful to test resistant genotypes in fields infected by BaYMV. Any potential mutations should be verified through artificial inoculation [33].

## Data Availability

All data supporting the findings of this study are available within this article and the Supplementary Material published online.

## Supplementary Materials

The following supporting information can be downloaded at: https://www.mdpi.com/article/10.3390/genes15060697/s1, Figure S1: Visualization with Integrative Genomics Viewer (IGV) of the reads aligned with the *Barley yellow mosaic virus* (BaYMV) strain BaYMV-I reference genome (GeneBank: AB430765 and AB430766); Figure S2: Small-scale genome-wide association studies analysis reveals the sites of amino acid variations in the polyproteins; Table S1: Summary of the RNA-seq data and mapping results in Takanezawa; Table S2: Percentage identity/distance matrix of RNA1 and RNA2. Estimation of the divergence between nucleotide sequences of BaYMV is illustrated by distance matrix (upper block) percentage identity (lower block); Table S3: Percentage identity/distance matrix of RNA1 and RNA2. Estimation of the divergence between amino acid sequences of BaYMV are illustrated by distance matrix (upper block) percentage identity; Table S4: (a) Correlated variations of RNA1 amino acids. Fields breaking *rym3* shaded in gray. (b) Correlated variations of RNA2 amino acids. Fields breaking *rym3* shaded in gray.
