# Supplementary material for: Identification of Barley yellow mosaic virus Isolates Breaking rym3 Resistance in Japan
Source: Genes (Basel). 2024 May 27;15(6):697. doi: 10.3390/genes15060697 (PMC11203024; doi:10.3390/genes15060697)
Supplement: Supplementary file 1 [file genes-15-00697-s001.zip › Supp.Figure S2_re2-submission.pptx]

## Slide 1
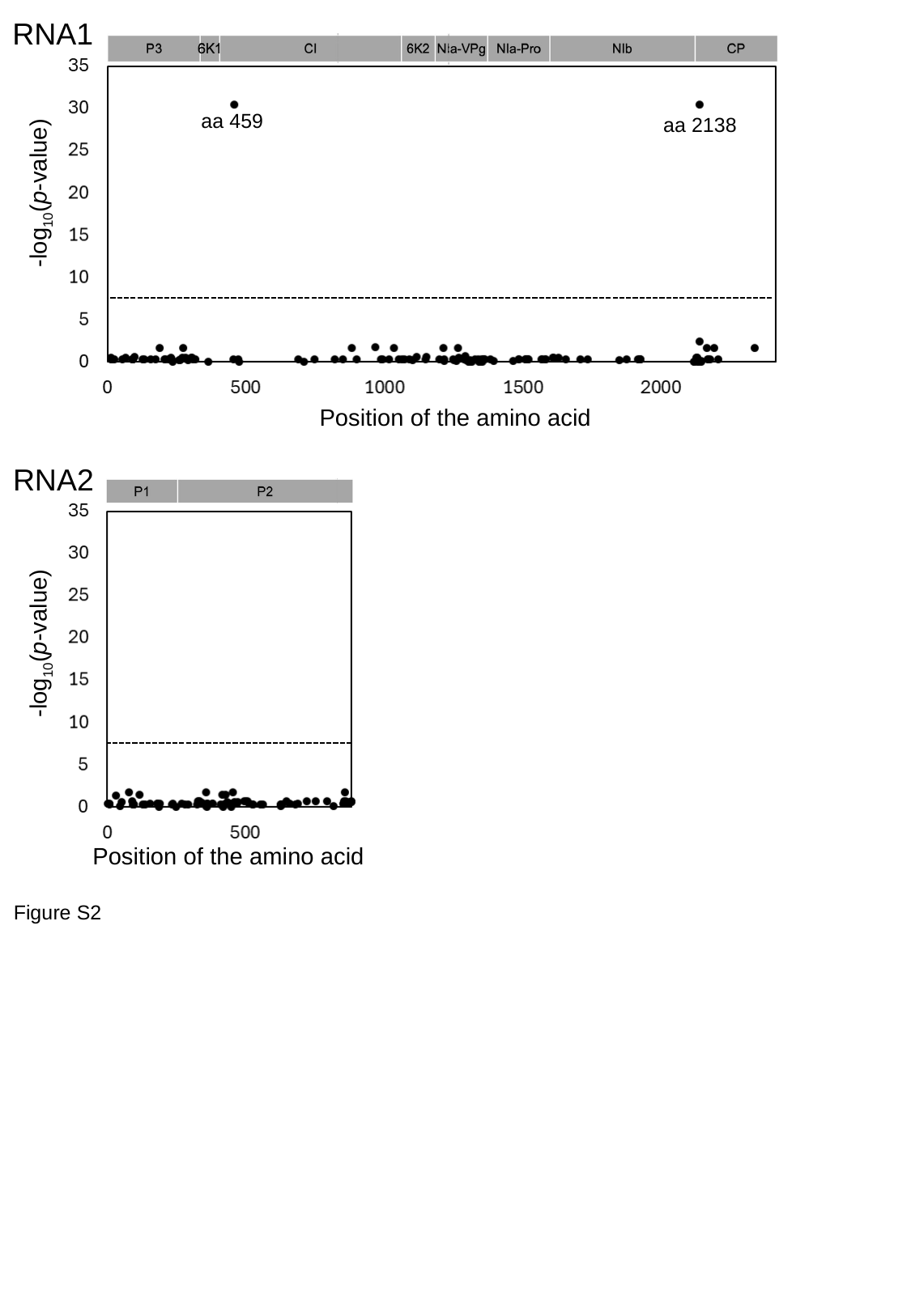

RNA1
aa 2138
-log10(p-value)
Position of the amino acid
aa2138
aa 459
RNA2
-log10(p-value)
Position of the amino acid
Figure S2
