# Supplementary figures and images for: Identification of Barley yellow mosaic virus Isolates Breaking rym3 Resistance in Japan
Source: Genes (Basel). 2024 May 27;15(6):697. doi: 10.3390/genes15060697 (PMC11203024; doi:10.3390/genes15060697)

## Slide 1
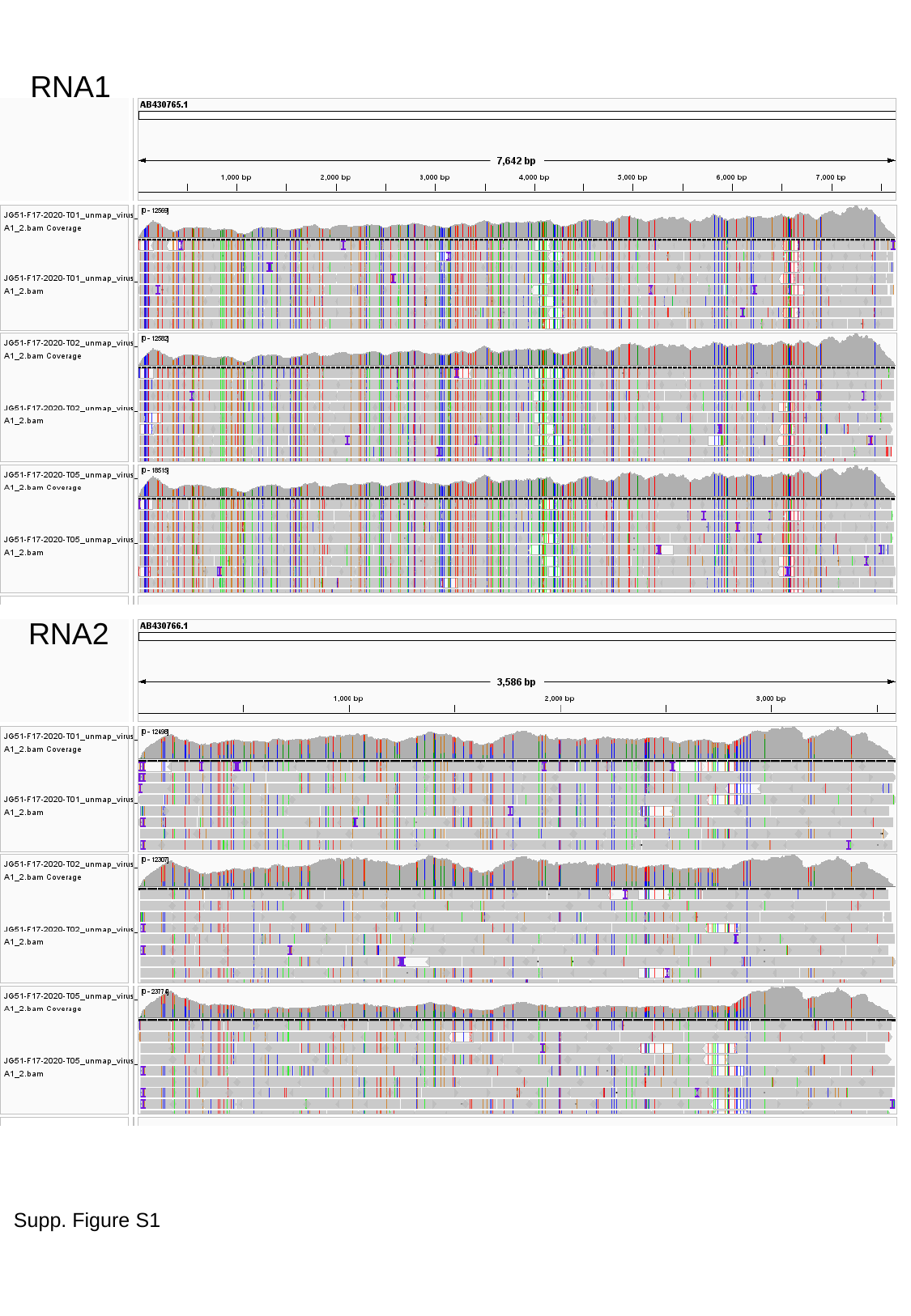

RNA1
RNA2
Supp. Figure S1

Supplement: Supplementary file 1 [file genes-15-00697-s001.zip › Supp.Figure S1_re2-submission.pptx]
